# Characterization of *Staphylococcus lugdunensis* biofilms through ethyl methanesulfonate mutagenesis

**DOI:** 10.3934/microbiol.2024038

**Published:** 2024-10-17

**Authors:** McKenna J. Cruikshank, Justine M. Pitzer, Kimia Ameri, Caleb V. Rother, Kathryn Cooper, Austin S. Nuxoll

**Affiliations:** 1 Department of Biology, University of Nebraska at Kearney, Kearney, NE, United States; 2 School of Interdisciplinary Informatics, College of Information Science and Technology, University of Nebraska at Omaha, Omaha, NE, USA

**Keywords:** *Staphylococcus lugdunensis*, biofilms, staphylococci, EMS mutagenesis

## Abstract

*Staphylococcus lugdunensis* is a coagulase-negative species responsible for a multitude of infections. These infections often resemble those caused by the more pathogenic staphylococcal species, *Staphylococcus aureus*, such as skin and soft tissue infections, prosthetic joint infections, and infective endocarditis. Despite a high mortality rate and infections that differ from other coagulase-negative species, little is known regarding *S. lugdunensis* pathogenesis. The objective of this study is to identify the essential factors for biofilm formation in *S. lugdunensis*. *S. lugdunensis* was mutagenized through ethyl methanesulfonate (EMS) exposure, and the individual cells were separated using a cell sorter and examined for biofilm formation at 8 hr and 24 hr timepoints. Mutations that resulted in either increased or decreased biofilm formation were sequenced to identify the genes responsible for the respective phenotypes. A mutation within the *S. lugdunensis* surface protein A (*slsA*) gene was common among all of the low biofilm formers, thus suggesting that high expression of this protein is important in biofilm formation. However, other mutations common among the mutants with decreased biofilm formation were in the putative divalent cation transport gene, *mgtE*. Conversely, a mutation in the gene that codes for the von Willebrand factor binding protein, *vwbl*, was common among the mutants with increased biofilm formation. Following proteinase K treatment, a significant dispersal of the *S*. *lugdunensis* biofilm matrix occurred, thus confirming the presence of primarily protein-mediated biofilms; this is in agreement with previous *S. lugdunensis* studies. Additionally, all low biofilm formers exhibited decreased protein levels (1.95–2.77 fold change) within the biofilm matrix, while no difference was observed with extracellular DNA (eDNA) or polysaccharides. This study presents a unique methodology to identify genes that affect biofilm formation and sheds light on *S. lugdunensis* pathogenesis.

## 1. Introduction

*Staphylococcus lugdunensis*, which is a coagulase-negative staphylococcal species (CoNS), can often be found on the human skin as normal microbiota [1]. While CoNS are not typically classified as aggressive human pathogens, *S. lugdunensis* is unique in that it exhibits virulence characteristics and infection patterns closer to that of the major human pathogen *Staphylococcus aureus*, with infections from minor skin and soft tissue infections to more serious and often life-threatening conditions such as infective endocarditis, prosthetic joint infections, and sepsis [2]. Retrospective clinical studies have found that this unique CoNS had an approximate global mortality rate of 39% in association with heart valve lesions, while endocarditis caused by *S. aureus* was estimated to have a global mortality rate closer to 20% [3],[4].

One of the primary ways that *S. lugdunensis* mediates infections similar to those caused by *S. aureus* is through biofilm formation [2]. These multicellular communities of bacteria are bound together by a matrix formed by biological materials such as polysaccharide intercellular adhesion (PIA), extracellular DNA (eDNA), and proteins [5]. This matrix produced by the bacteria is protective against antibiotics, disinfectants, and the host immune system, which makes biofilm-associated infections extremely difficult to treat [6],[7]. Following biofilm maturation, quorum sensing leads to the dissociation of the biofilm and the subsequential spreading to other areas of the host, thus potentially causing a life-threatening systemic infection [8].

While *S. lugdunensis* shares the ability to cause severe biofilm-mediated infections with *S. aureus*, the biofilms of *S. lugdunensis* remain relatively uncharacterized. The objective of this study is to further characterize these biofilms. Specifically, genetic factors important for *S. lugdunensis* biofilm formation are examined through ethyl methanesulfonate (EMS) mutagenesis, followed by biofilm screening. One gene of interest is the *S. lugdunensis* surface protein A (*slsA*) gene. SlsA is a putative LPXTG cell-wall anchored protein, which is a class of proteins commonly associated with biofilm formation in staphylococci [9]. While the *slsA* gene was associated with biofilm formation in the EMS screen, there are several other mutations in the low biofilm formers, including mutations in a putative divalent cation transport protein gene, *mgtE*. Among the high biofilm formers, a common mutation was found in the gene that encodes the von Willebrand factor binding protein, *vwbl*. These results identified three genes involved in biofilm formation in *S. lugdunensis*. Furthermore, the characterization of the biofilm matrix in low biofilm formers revealed a decrease in the overall protein composition, whereas the eDNA and polysaccharide concentrations remained unaltered.

## 2. Materials and methods

### 2.1. Strains and growth conditions

*S. lugdunensis* strain N920143 was used in all experiments [10]. Methicillin susceptible *S. aureus* strain HG003 and *Staphylococcus epidermidis* strain 1457 were used as controls in the biofilm dispersal assays [11],[12]. Unless otherwise stated, growth occurred at 37 °C, 225 rpm, and in 3 mL Tryptic Soy Broth (TSB) in a 14 mL snap cap tube.

### 2.2. Ethyl methanesulfonate mutagenesis

Following a 1:100 dilution, the overnight cultures were grown for 4.5 hr to mid-exponential phase (OD_600_ = ~ 3.5). 500 µL of the culture, 250 µL 125 mM HEPES, and 15 µL of EMS (100 µg/mL) were added to a 1.5 mL centrifuge tube, incubated for 45 min at 37 °C, washed with 1% NaCl, and then plated on either TSA or TSA containing rifampicin (10 µg/mL) to determine if mutagenesis occurred. To determine the resistance frequency following mutagenesis, the number of colonies recovered on the TSA containing rifampicin was divided by the number of colonies recovered on the TSA. Following mutagenesis, the cultures were sorted (Sony SH800) onto a rectangular TSA plate at one event per spot with a total of 1344 cells, and were subsequently incubated at 37 °C for 24 hr. The experiments were performed in triplicate and the significance was determined using a student's t-test, P ≤ 0.05.

### 2.3. High-throughput biofilm screen

Overnight cultures of *S. lugdunensis* were diluted 1:100 in 100 µL TSB within a 96-well tissue culture treated plate (Costar #3628) and were statically incubated at 37 °C for either 8 hr (immature biofilm) or 24 hr (mature biofilm). After the biofilms formed, the non-adherent cells were removed by washing with 1% NaCl and stained with 50 µL of crystal violet dye. The biofilms were stained at room temperature for 2 min. Following staining, the biofilms were washed with distilled water to remove any excess stain. To quantify the biofilm matrix, 100 µL ethanol was used to solubilize the biofilms and the absorbance was measured at 600 nm using a BioTek Synergy H1 plate reader.

### 2.4. Growth rate and yield analysis

Growth of the mutagenized colonies was assessed by diluting the overnight cultures 1:100 in 100 µL TSB in a 96-well microtiter plate. Growth was monitored in a microplate reader (BioTex Synergy H1, serial number 170822D) with continuous shaking at 37 °C for 24 hr. The absorbance was read at 600 nm every 30 min and growth curves were determined for each mutant. The experiments were performed in triplicate and the standard deviation is represented.

### 2.5. Sequencing and genomic analysis

DNA (100 ng) from isolates that were found to have high and low biofilms compared to the parent strain were sent to the genomics core facility at the University of Nebraska Medical Center to identify nucleotide changes. Genomic DNA libraries were prepared using the Nextera XT Library Preparation Kit (Illumina) according to manufacturer instructions. A pool of 12 indexed libraries was sequenced using the Illumina NextSeq 550 system using a mid-output flow cell with a 75 paired-end run. vCompressed fastq files were downloaded and stored in a static read-only archive. FastQC, version 0.11.7, was used to guide the quality control and data cleaning. Trimmomatic, version 0.36, was used to clip the adapter sequences and to trim low-quality reads, primarily at the 3′ end of the reads. BWA-MEM, version 0.7.17, was used to align the sequencing reads against the reference genome (*S. lugdunensis* N920143 complete genome, taxnomomy ID 1034809, downloaded from the National Institutes of Health Genome Browser). Then, *Samtools*, v.1.9, was used to sort convert the SAM to BAM files. Then, Picard MarkDuplicates, version 2.9, was used to locate and tag the duplicate reads, and the quality was assessed using Qualimap, v.2.2.1. Variant calling was performed with Freebayes, v.0.9.10, where the output included all the predicted variants (reference + observed allele) and a brief report on their quality scores. VCFTools was used to filter the quality scores (QUAL) and the depth of each to a QUAL > 1000 and a DP > 100. The intersection of variants between high biofilm formers was found using the bcftools (version 1.8) isec function, as was the intersection of the variants from the low biofilm formers. SnpEff, v.4.3, was used to annotate the variants and calculated the effects they produced on the known genes. The inputs were predicted variants in the variant call format (VCF).

### 2.6. Dispersal of biofilms

*S. lugdunensis* and low biofilm mutants were grown overnight, diluted 1:100 in 100 µL TSB in a 96-well microtiter plate (Costar #3628), and grown for 24 hr at 37 °C. Following incubation, the biofilms were washed with PBS and statically incubated at 37 °C in either 100 µg/mL proteinase K in proteinase K buffer (20 mM HCl Tris, 100 mM NaCl, pH 7.5) or in proteinase K buffer alone. The medium was removed, and the strains were washed again with 1% NaCl. The biofilms were stained with crystal violet dye and were statically incubated for 1 min. The stain was removed by washing with 1% NaCl twice. 100 µL ethanol was added to the wells to solubilize the biofilms, and the absorbance was read at 600 nm using a BioTek Synergy H1 plate reader. The experiments were performed in triplicate and the statistical significance was determined using a two-way ANOVA followed by a *post hoc* Sidak's test (p ≤ 0.05).

### 2.7. Staining of biofilm matrix

The overnight cultures of *S. lugdunensis* were diluted 1:100 in 100 µL TSB within a 96-well tissue culture-treated plate (Costar #3628) and were statically incubated at 37 °C for 24 hr. Following incubation, the biofilms were washed with 100 µL 1% NaCl and stained with either 100 µL concanavalin A (50 µg/mL for 60 min, ex488/em545, MP Biomedicals), 4′,6-diamidino-2-phenylindole-DAPI (1 µg/mL for 15 min, ex350/em465, Invitrogen), or SYPRO ruby biofilm matrix stain (1:1 ratio for 30 min, ex450/em610, Invitrogen). Following incubation with the dyes, the biofilms were washed with 100 µL NaCl and their fluorescence were measured using a BioTek Synergy H1 plate reader at each stain's respective excitation/emission spectrum. The experiments were performed in triplicate and the statistical significance was determined using a one-way ANOVA followed by a Dunnett's multiple comparison test (p ≤ 0.05).

## 3. Results

### 3.1. Creating a mutagenesis library for high throughput screening

**Figure 1.**
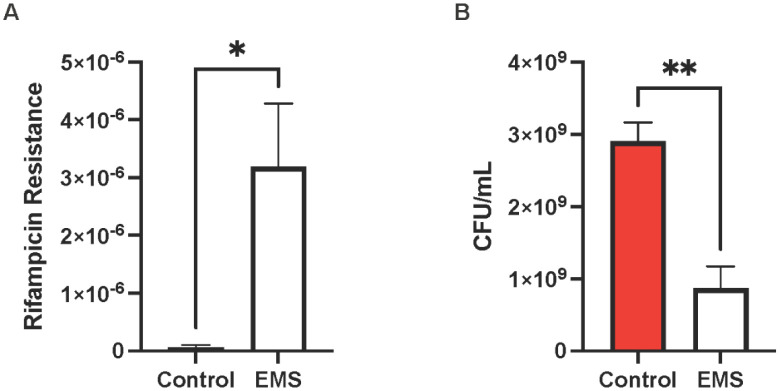
Rifampicin resistance frequency following EMS mutagenesis. (A) *S. lugdunensis* cultures were treated with EMS for 45 min or in HEPES buffer in the absence of EMS (control) and then plated on either TSA or TSA/Rifampicin plates to determine if mutagenesis occurred. (B) Following mutagenesis, the surviving cells were plated on TSA to determine the bacterial viability following EMS mutagenesis. Experiments were performed in triplicate, with error bars representing the standard deviation, and the significance was determined using a student's t-test, P < 0.05.

To identify the factors involved in *S. lugdunensis* biofilm formation, EMS mutagenesis was performed in *S. lugdunensis*. EMS creates random mutations throughout the genome following guanine alkylation. To confirm if mutagenesis occurred, the rifampicin resistance frequency was determined in cultures treated with EMS (Figure 1A; p = 0.0402). The rifampicin resistance frequency in untreated cultures was 6.9 × 10^−8^. Following EMS treatment, the frequency increased to 3.2 × 10^−6^, thus indicating a significant increase in mutagenesis. Increased mutagenesis is likely to result in some loss of viability. To ensure that the cells remained viable, the survival was measured following EMS treatment (Figure 1B; p = 0.0051). As expected, there was a reduction in the number of viable cells following treatment; however, the majority of the population (1 × 10^9^ cells/mL) remained viable.

### 3.2. High throughput screening for altered biofilm formation

**Figure 2.**
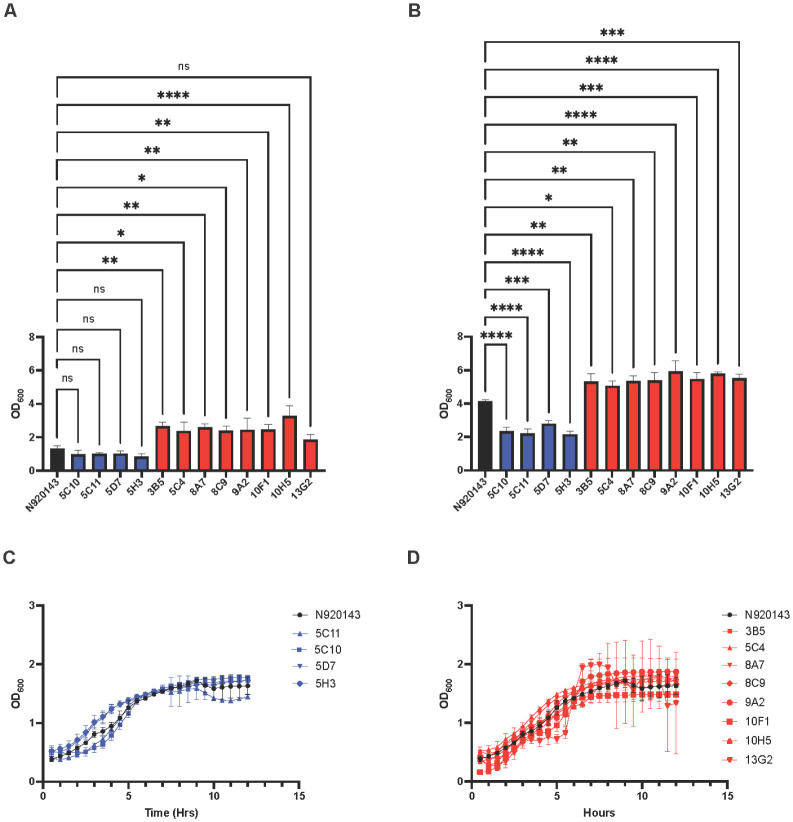
Biofilm formation and growth rates of EMS mutants. Cultures of EMS mutants were grown for either (A) 8 hr or (B) 24 hr in a 96-well plate. The biofilms were stained with crystal violet, solubilized, and analyzed using a plate reader. The biofilm experiments were performed in triplicate with error bars representing the standard deviation, and the significance was determined through a one-way ANOVA followed by a Dunnett's multiple comparison test (*P < 0.05, **P < 0.005, ***P < 0.0005, ****P < 0.0001). To determine the growth rate, EMS cultures were monitored in a microplate reader for 24 hr. The absorbance was read at 600 nm every 30 min and growth curves were determined for (C) the low biofilm formers and (D) the high biofilm formers. The growth for each mutant was measured in triplicate and the error bars represent the standard deviation.

Following the mutagenesis of *S. lugdunensis* cultures, individual cells were sorted onto rectangular TSA plates at one event per spot. Doublets were excluded by gating on the forward scatter area vs the forward scatter height. Biofilm formation was analyzed in 1344 mutagenized cells for either increases or decreases compared to the parent strain. We reasoned that increases in biofilm formation may be challenging to assess in mature biofilms, and that decreases in biofilm formation that resulted from decreased growth rates would be more apparent in immature biofilms. Therefore, to determine EMS mutants that led to either increased or decreased biofilm formation, screening was performed at both 8 hr (immature biofilms) and 24 hr (mature biofilms). Mutants exhibiting altered biofilm formation at either 8 hr or 24 hr were selected for a further analysis (Figure 2A,B). To ensure that the mutations were affecting biofilm formation and were not an artifact of mutations that altered the metabolism, EMS mutants were monitored for any changes in either the growth rate or the growth yield (Figure 2C,D). Mutants that exhibited significant alterations in either the growth rate or the growth yield compared to the parent strain, N920143, were excluded from further analyses, as the biofilm defects were likely a result of changes in the growth rates. Following a growth rate analysis, four low biofilm formers (5C10, 5C11, 5D7, 5H3) and eight high biofilm formers (3B5, 5C4, 8A7, 8C9, 9A2, 10F1, 10H5, 13G2) were selected for a sequence analysis to identify the mutations responsible for the respective biofilm phenotypes. While it was initially predicted that increases in biofilm formation wouldn't be apparent in mature biofilms, a significant increase in biofilm formation was apparent in all high biofilm formers at 24 hr. Therefore, low biofilm and high biofilm formers were designated by having a statistically significantly decrease or increase in biofilm formation compared to the N920143 parent strain in a mature biofilm as measured by crystal violet staining.

### 3.3. Identifying mutations responsible for altered biofilm phenotype

Sequencing data was used to search for non-synonymous variants in high and low biofilm formers. Data was high quality and 97% of reads were retained after pre-processing the sequencing data with Trimmomatic. The GC content was reported at 34%, which is in line with the reported *S. lugdunensis* genome GC content of 33.7953%. There was a high coverage of the genome, with an average coverage range of 130 to 290. The average read mapping after aligning the sequencing reads to the reference genome was 99.94%, with a range of 99.91–99.95% mapping across all the samples. The data was deposited with links to BioProject accession number PRJNA1140638 in the NCBI BioProject database (https://www.ncbi.nlm.nih.gov/bioproject/1140638).

SnpEff describes variant impact definitions as HIGH, MODERATE, LOW, or MODIFIER based on the anticipated impact on the analyzed protein or gene product. For this study, we focused on variants annotated as HIGH (disruptive impact or stop gain mutations) and MODERATE (some possible impact or missense variants). Among the four strains that exhibited a decreased biofilm formation, there were two common variants. A mutation in the *slsA* gene and the *mgtE* gene resulted in a missense and a frameshift mutation, respectively (Table 1). A common mutation among the eight high biofilm formers was found in the *vwbl* gene, which resulted in a missense mutation (Table 2).

**Table 1.** Common variants in all 4 low biofilm isolates.

Chromosome Position	Ref.	ALT	SnpEff Annot.	Gene Id	Transcript Id/Protein	Function	Type	Sequence
1957808	TTAT	TT	HIGH	SLUG_18540 (*mgtE*)	CCB54356.1	Putative divalent cation transport protein	Frameshift	1957803..1959080
372929	CCAA	GGAA	MOD.	SLUG_03480 (*slsA*)	CCB52765.1	Putative LPXTG cell wall-anchored protein	Missense	368457..374249

**Table 2.** Common variants in all 8 high biofilm isolates.

Chromosome Position	Ref.	ALT	SnpEff Annot.	Gene Id	Transcript Id/Protein	Function	Type	Sequence
2465879	CTCTTG	TTCCTT	MOD.	SLUG_23290 (*vwbl*)	CCB54854.1	Von Willebrand factor-binding protein precursor	Missense	2464430..2470009

### 3.4. Characterization of S. lugdunensis biofilms

**Figure 3.**
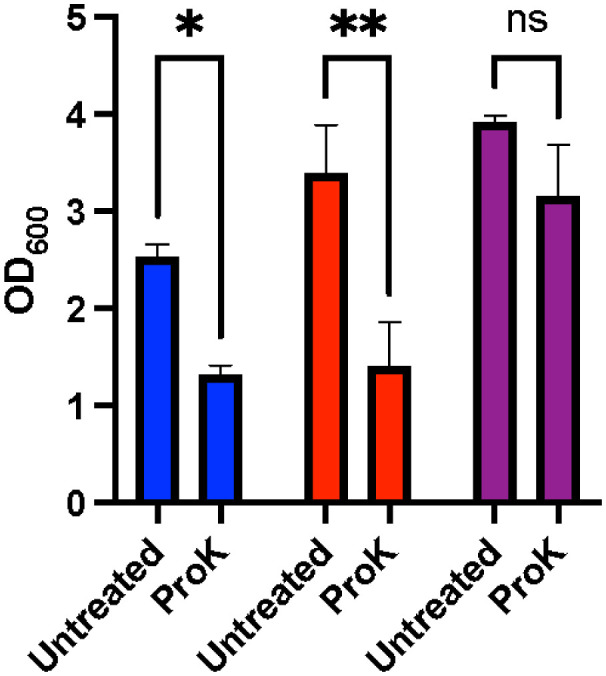
Proteinase K dispersal of staphylococcal biofilms. *S*. *aureus* (blue), *S. lugdunensis* (red), and *S. epidermidis* (purple) biofilms were treated with either proteinase K or buffer alone. Following treatment with proteinase K, the biofilms were stained with crystal violet and analyzed for dispersal. *S. lugdunensis and S. aureus biofilms* were dispersed in the presence of proteinase K, thus indicating a predominantly protein-mediated biofilm. The experiments were performed in triplicate, with error bars representing the standard deviation, and the significance was determined through a two-way ANOVA followed by a Sidak's multiple comparison test (*P < 0.05, **P < 0.005).

While two unique mutations were identified to be present among all the low biofilm formers and one unique mutation was identified among all the high biofilm formers, *slsA* was of a particular interest, as surface proteins that are anchored to the cell wall by an LPXTG motif have been shown to be important for biofilm formation in closely related species, namely *S. aureus* and *S. epidermidis* [13],[14]. Based on these sequencing results, it was hypothesized that the *S. lugdunensis* biofilms were primarily protein mediated. To determine if this was indeed the case, the biofilms were treated with proteinase K. Following the proteinase K challenge, the *S. aureus* and *S. lugdunensis* biofilms were significantly disrupted; alternatively, there was no significant decrease in *S. epidermidis* (Figure 3; p = 0.0487 and p = 0.0049, respectively). These findings support the sequence analysis which identified a gene (*slsA)* that coded for a LPXTG cell-wall anchored protein as a factor that could be involved with *S. lugdunensis* biofilm formation.

**Figure 4.**
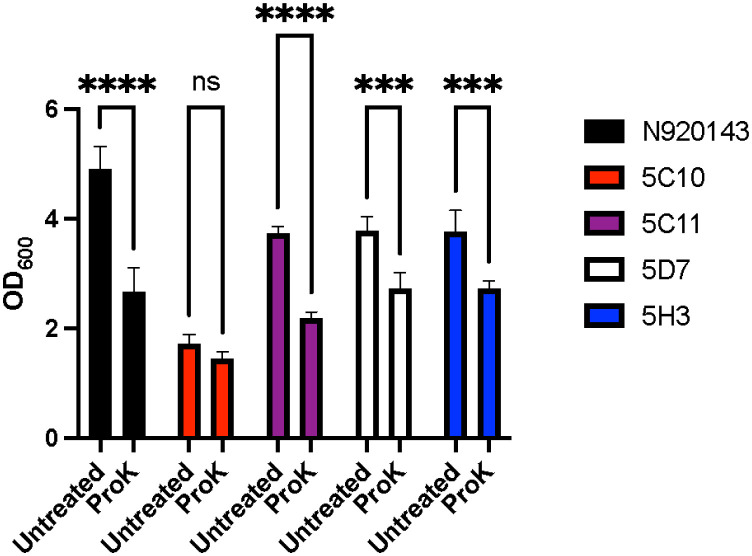
Biofilm dispersal of EMS mutants. *S. lugdunensis* N920143 and the low biofilm former mutants' biofilms were treated with either proteinase K or buffer alone. Following treatment with proteinase K, the biofilms were stained with crystal violet and analyzed for the dispersal of biofilm matrix. Proteinase K treatment resulted in a further dispersal of the biofilms in three of the EMS mutants in addition to the parent N920143 strain. The experiments were performed in triplicate and the significance was determined through a two-way ANOVA followed by a Sidak's multiple comparison test (***P < 0.0005, ****P < 0.0001). The error bars are representative of the standard deviation.

Based on the findings in the biofilm dispersal assay, we reasoned that the low biofilm formers from EMS mutagenesis would not be affected by the proteinase K treatment, as the SlsA protein was already disrupted in these mutants. Following proteinase K dispersal, the wild-type and three of the low biofilm formers, 5C11, 5D7, and 5H3, exhibited a significant dispersal of the biofilm matrix, while there was no change in 5C10 (Figure 4). These results suggest that other proteins alongside SlsA are important for *S. lugdunensis* biofilm formation.

**Figure 5.**
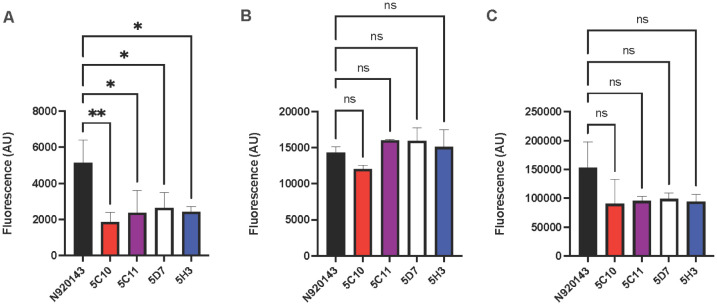
Low biofilm formers have decreased protein within the biofilm matrix. Mature biofilms were stained with either SYPRO ruby biofilm matrix stain (Figure 5A), DAPI (Figure 5B), or concanavalin A (Figure 5C). Following incubation with the dyes, the biofilms were washed to remove the excess dye, and the fluorescence was measured at ex450/em610, ex350/em465, and ex488/em545, respectively. The EMS mutants exhibited decreased staining with SYPRO, but no significant difference was observed with either DAPI or conA staining. The experiments were performed in triplicate, with error bars representing the standard deviation, and the significance was determined with a one-way ANOVA followed by a Dunnett's multiple comparison test (*P < 0.05, **P < 0.005).

Based on the unexpected findings that the proteinase K treatment further reduced bacterial burden, the biofilm composition was analyzed to determine if the low biofilm formation in the EMS mutants was a result of either decreased proteins, eDNA, polysaccharide, or a combination of those factors. As expected, the four EMS mutants with a low biofilm formation all exhibited a significant decrease in protein concentrations within the biofilm matrix (Figure 5A). When examining the composition of eDNA in the biofilm matrix, no difference was observed between the wild-type and the low biofilm formers (Figure 5B). Interestingly, when examining the polysaccharides within the biofilm matrices, the four EMS mutants showed decreased polysaccharide contents compared to the N920143 wild-type strain, albeit not significant decreases (Figure 5C). These findings support the notion that *S. lugdunensis* forms protein-mediated biofilms and *slsA* is likely involved in this process.

## 4. Discussion and conclusions

*S. lugdunensis* is responsible for infections ranging from skin and soft tissue infections to biofilm mediated infections including prosthetic joint infections and endocarditis. Among all bacterial infections, it is estimated that biofilms are associated with 60% to 80% of infections [15]. Biofilms are problematic for not only the host's immune system, but also contribute to antibiotic therapy failure [6],[16],[17]. While *S. lugdunensis* is classified as a coagulase-negative staphylococci, the clinical features of this pathogen more closely resemble *S. aureus* [18]. Despite the pathogenic nature and clinical importance, relatively little is known regarding the factors that are required for *S. lugdunensis* biofilm formation.

Through ethyl methanesulfonate mutagenesis coupled with cell sorting, we identified several factors that are likely to impact biofilm formation in *S. lugdunensis*. A common mutation in the *slsA* gene and the *mgtE* gene were found among the low biofilm formers (Table 1), while a common mutation in the *vwbl* gene was found among the high biofilm formers (Table 2). The mutation in the *slsA* gene that resulted in a decreased biofilm formation was not surprising, as it encodes a surface protein that is anchored to the cell wall through an LPXTG motif. Similar surface proteins have been found to play roles in biofilm formation in other staphylococcal species [13]. SasC, SasG, and Bap in *S. aureus* and Aap in *S. epidermidis* are surface proteins that are important for biofilm formation that contain an LPXTG motif [19]–[22]. Additionally, this is in agreement with previous work which found that *S. lugdunensis* biofilms were primarily protein mediated and not polysaccharide mediated [23],[24].

In this study, all low biofilm formers which were identified through EMS mutagenesis contained mutations in both *mgtE* and *slsA*, thus suggesting that both mutations were important for the observed phenotype. The *mgtE* encodes putative divalent cation transport proteins. Divalent cations are involved in cell-to-cell adhesion due to interactions with teichoic acids in the cell wall; additionally, divalent cation concentrations have been shown to affect extracellular polysaccharide and proteinaceous materials in biofilms [25]–[27]. It stands to reason that the observed decrease in biofilm formation may be a result of both the *slsA* and *mgtE* mutations that altered the composition of the biofilm matrix. However, a number of individual point mutations existed within each individual strain; therefore, one cannot exclude the possibility of other mutations contributing to these biofilm phenotypes. It is important to note that no mutations outside of *slsA* and *mgtE* were common among the four low biofilm formers; additionally, they were absent from the high biofilm formers mutants. Additional point mutations could explain the findings observed with 5C10 not showing a further reduction in biofilm formation following proteinase K dispersal (Figure 4). While the protein composition was significantly decreased in all four of the low biofilm formers, 5C10 exhibited a stronger phenotype compared to the other EMS mutants, thus indicating that other point mutations likely impact the biofilm formation (Figure 5). In addition to the decrease in proteins within the biofilm matrix, there was a slight reduction in the observed polysaccharides within the mutants (Figure 5), which could be the result of the mutation in *mgtE*.

Interestingly, a mutation within *vwbl*, which codes for the von Willebrand factor binding protein, resulted in an increased biofilm formation. Recently, work in *Mycobacterium tuberculosis* also implicated a protein that contained the von Willebrand factor domain in biofilm formation, which supports the finding that *vwbl* may be involved in biofilm formation [28]. In *S. aureus*, a number of studies highlighted the importance of the von Willebrand factor binding protein during *in vivo* adherence and the establishment of infection [29]–[32]. Fitting with these findings, a mutation of *vwbl* in *S. lugdunensis* resulted in reduced virulence within a rat endocarditis infection model [33].

The staphylococcal species are one of the primary causes of biofilm-mediated indwelling medical device infections. Many studies have aimed to understand the mechanism of biofilm formation in other staphylococcal species, such as *S. aureus* and *S. epidermidis*; however, the factors responsible for biofilm formation in *S. lugdunensis* are less studied. The last couple of decades have led to a better appreciation for the clinical importance of *S. lugdunensis*; however, there is much that is unknown regarding the pathogenesis of this organism. While this study identified mutations that were common among four mutants with reduced biofilm formation and one mutation that resulted in an enhanced biofilm formation, genetic deletions of these genes are needed to confirm their role in biofilm formation in *S. lugdunensis*. While *slsA*, *mgtE*, and *vwbl* were the only mutations identified in this study, many single nucleotide polymorphisms exist in each mutant sequence and could impact the observed biofilm phenotypes. Additionally, EMS mutagenesis is restricted single nucleotide polymorphisms (SNPs), many of which could be silent or result in only a partial loss of protein function, thus leading to a number of genes involved with biofilm formation going unidentified. Despite these limitations, this work highlighted common mutations among all high biofilm formers, as well as those present among all low biofilm formers, thus suggesting the importance of these factors to *S. lugdunensis* biofilm formation Additional experimentation is required to further understand the mechanism of biofilm formation in *S. lugdunensis*.

## Use of AI tools declaration

The authors declare they have not used Artificial Intelligence (AI) tools in the creation of this article.
